# Maternal Metformin Exposure Induces Gut Microbial Shifts in Non-Diabetic Dams and Sex-Stratified Changes in Mouse Offspring

**DOI:** 10.3390/biom16071017

**Published:** 2026-07-12

**Authors:** Dat Da Ly, Kate Phuong-Nguyen, Trang T. T. Truong, Bryony A. McNeill, Kathryn Aston-Mourney, Leni R. Rivera

**Affiliations:** 1 School of Medicine, The Institute for Mental and Physical Health and Clinical Translation, Deakin University, Geelong, VIC 3216, Australia; s223518527@deakin.edu.au (D.D.L.); katephuongnguyen.nguyen@unimelb.edu.au (K.P.-N.); t.truong@deakin.edu.au (T.T.T.T.); bryony.mcneill@deakin.edu.au (B.A.M.); k.astonmourney@deakin.edu.au (K.A.-M.); 2 School of Agriculture, Food and Ecosystem Sciences, Human Nutrition, University of Melbourne, Parkville, VIC 3010, Australia

**Keywords:** maternal, metformin, gut microbiota, non-diabetic mice, offspring, 16S rRNA

## Abstract

Beyond being one of the most widely prescribed medications for type 2 diabetes, metformin has been increasingly used during pregnancy for gestational diabetes and polyendocrine metabolic ovarian syndrome. While it is considered safe for pregnancy and lactation, metformin is known to induce gut microbial changes in non-pregnant individuals with type 2 diabetes, and maternal and offspring microbial alterations are known to influence offspring development. Therefore, understanding whether metformin drives microbiota changes beyond the confounding effects of maternal hyperglycaemia holds significant implications for maternal–foetal health. In this study, non-diabetic C57BL/6 mouse dams received either control or metformin treatment (5 mg/mL) in drinking water during pregnancy and lactation. Microbial profiling from faecal samples of dams at gestational day 15 and offspring at 6 weeks of age was analysed using 16S rRNA sequencing and an R-based pipeline. Metformin positively restructured dominant taxa associated with healthy pregnancy in dams, while inducing broad taxonomic changes in males and Bacteroidetes-dominant shifts in female offspring. Overall, maternal metformin treatment promoted short-chain fatty acid-producing taxa while suppressing potentially opportunistic bacteria, without compromising community richness. Further studies are required to elucidate the functional mechanisms underlying these shifts and their long-term implications for maternal–foetal health.

## 1. Introduction

Type 2 diabetes (T2D), defined as impaired glucose homeostasis due to insulin resistance and/or inadequate insulin secretion [1], affected over 500 million people worldwide in 2021 [2]. It is strongly associated with microvascular and macrovascular complications and is closely linked to the global obesity epidemic [1,3]. Following lifestyle and dietary interventions, metformin remains the most widely prescribed first-line pharmacological therapy, as it is safe and effective, with approximately 150 million users globally as of 2022 [4].

Metformin is also recommended as a first-line treatment for diabetes in pregnancy by major clinical guidelines, including those from the Endocrine Society, International Federation of Gynecology and Obstetrics, National Institute for Health and Care Excellence, Hong Kong College of Obstetricians and Gynaecologists, and Queensland Health [5,6,7,8]. Beyond its approved use in diabetes, metformin is increasingly investigated and used in off-label contexts [9], including polyendocrine metabolic ovarian syndrome (PMOS; previously referred to as PCOS—polycystic ovary syndrome) [10,11,12,13], prediabetes [14,15], chronic kidney disease [16,17], metabolic dysfunction-associated steatotic liver disease (MASLD; previously termed non-alcoholic fatty liver disease) [18,19], obesity [20,21,22], dementia [23,24], and cancer prevention [25,26,27]. In reproductive medicine, metformin is widely used during pregnancy in women with PMOS, particularly for the management of anovulatory infertility [28,29]. It has also been investigated in obese, non-diabetic pregnancies; however, randomised trials have shown limited improvements in neonatal outcomes, likely reflecting heterogeneity in maternal insulin resistance, suboptimal adherence, and limited long-term follow-up [30,31,32]. As a result, the perinatal effects of maternal metformin exposure in non-diabetic pregnancies remain unclear.

Metformin lowers glucose through coordinated effects on hepatic gluconeogenesis, peripheral glucose uptake, and intestinal glucagon-like peptide-1 secretion [33,34]. Increasing evidence suggests that the gut microbiota may act as an additional mediator of its effects. Metformin promotes the growth of beneficial taxa, including *Akkermansia* and short-chain fatty acid (SCFA)-producing bacteria, and can partially reverse T2D-associated dysbiosis [35,36,37,38]. However, its microbiota-mediated actions remain less well defined than its established metabolic effects, particularly in pregnancy. The maternal gut microbiota plays a critical role in neonatal outcomes and early-life development [39,40]. Dysbiosis during pregnancy has been associated with alterations in infant neurodevelopment, immune maturation, and the establishment of the gut–brain axis [41,42,43,44]. In animal models of high-fat-diet-induced metabolic dysfunction, maternal metformin treatment improves gut dysbiosis, accompanied by sex-specific alterations in offspring microbiota [45]. In humans, metformin treatment in gestational diabetes mellitus (GDM) has been shown to increase Proteobacteria and Enterobacteriaceae, which are inversely associated with postprandial glycaemia and maternal weight gain [46].

While these findings suggest that metformin influences the maternal gut microbiota, causality remains unclear—specifically, whether microbial changes reflect direct effects of the drug or secondary responses to improved glycaemic control. This distinction is difficult to resolve in diabetic models. Therefore, the present study examines the effects of metformin on the gut microbiota in a non-diabetic pregnant mouse model and their offspring, enabling the separation of direct microbiota-modulating effects from indirect metabolic influences and addressing a key gap in understanding metformin’s action during pregnancy.

## 2. Materials and Methods

### 2.1. Animal Model

All animal procedures were performed in accordance with the guidelines of the Deakin University Animal Ethics Committee (VIC, Australia; Project ID: G23-2020; approval date: 11 March 2021) and the National Health and Medical Research Council. Non-diabetic C57BL/6 mice were purchased from the Animal Resources Centre (Perth, WA, Australia) at 7 weeks of age, couriered to Deakin University, and acclimated for one week before any intervention. Mice were maintained on a strict 12-hour light/dark cycle (lights turned on at 7 am and off at 7 pm), humidity at 40–70%, and a controlled temperature of 21 ± 3 °C, with ad libitum access to food and water. All mice were fed a standard chow diet.

### 2.2. Mating and Pregnancy

One 8- to 14-week-old female and one 8- to 30-week-old male mouse were housed together for mating (1:1 ratio). Mating was confirmed by visual inspection of a vaginal plug (denoted gestational day (GD) 0.5). If a vaginal plug was not detected after 3 days of mating, mice were still followed in the same manner until pregnancy was confirmed by monitoring maternal body weight gain and visual assessment of abdominal enlargement on approximately day 14. Body weight and blood glucose measurements and faecal collection were conducted every week. Mice exhibited the expected increase in body weight during pregnancy and maintained normal-range glycaemia, with no difference between treatment groups. Pregnant mice were housed individually with ad libitum access to a standard chow diet and randomly assigned to either regular sterile drinking water (control group: Ctrl) or metformin-added drinking water (metformin group: Met) from GD0.5 until postnatal day 21. Metformin (Stress Marq Biosciences, Victoria, BC, Canada) was completely dissolved in 1 L of regular sterile drinking water at the concentration 5 mg/mL. To avoid any unexpected effect of the litter size on nutrition supply during pregnancy and long-term metabolic outcomes, studies were only carried out on litters with 6–10 pups, which were then normalised to 6 mice on the day of birth. Any extra pups were humanely culled by decapitation. Following delivery, offspring remained with their mother until weaning at postnatal day 21. After weaning, dams were euthanised by slow-fill CO_2_ inhalation followed by cervical dislocation. Offspring were fed a standard chow diet, and, at 6 weeks of age, faecal samples were collected and mice humanely culled.

### 2.3. Analysis of Gut Microbiota Composition

#### 2.3.1. DNA Extraction and 16S rRNA Sequencing

DNA was extracted from faecal samples collected from dams on GD15 (*n* = 6/group), and from female and male offspring at 6 weeks of age (*n* = 8/group), using a QIAamp Fast DNA Stool Mini Kit (Qiagen Pty Ltd., Clayton, VIC, Australia), as per the manufacturer’s instructions. PCR amplification and 16S rRNA sequencing (variable region V3–V4) were performed by the Australian Genome Research Facility (AGRF). Forward primer for 16S (341F): CCTAYGGGRBGCASCAG; reverse primer (806R): GGACTACNNGGGTATCTAAT.

Raw paired-end 16S rRNA (V3–V4) reads generated by AGRF were processed using the QIIME2 [47] platform (version 2022.8). Reads were quality- and length-filtered using the Cutadapt plugin (default settings). The DADA2 [48] plugin was then applied for denoising and chimera removal to generate amplicon sequence variants (ASVs). Taxonomy was assigned using a naïve Bayes classifier against the Greengenes (13_8) [49] reference database, pre-clustered at 99% identity [50]. BLAST (version 2.9.0) searches were performed against the NCBI nt database to identify reference sequences similar to each ASV, and the top hit was assigned to the corresponding ASV. The resulting ASV feature table (BIOM format) and rooted phylogenetic tree were used as inputs for all downstream R-based analyses in phyloseq.

#### 2.3.2. Statistical and Microbial Analyses

Post-bioinformatics analyses of faecal microbiota were conducted using RStudio (version 2025.05.1+513). After removing one outlier (in the group of metformin-exposed male offspring) identified by the Mahalanobis distance squared (Supplementary Figure S1), data analyses included the assessment of changes in alpha (α) diversity, beta (β) diversity, relative abundance, and differential abundance. A minimum read count threshold of 4000 reads per sample was applied prior to analysis. Rarefaction was not applied; α- and β-diversity analyses were performed on unrarefied count data. To confirm robustness, β-diversity distances were also computed on relative abundance-transformed data; conclusions were unchanged across all metrics (Mantel r ≥ 0.96, 999 permutations). The differential abundance analysis used DESeq2 with poscounts size factor normalisation.

α-diversity (Shannon and Fisher indices) was calculated to evaluate microbial diversity within each sample using the estimate richness function in the phyloseq [51] package and visualised with boxplots via phyloseq and ggplot2 [52]. Statistical analysis was performed using the Wilcoxon test. β-diversity was assessed using Bray–Curtis dissimilarities and weighted/unweighted UniFrac and visualised through principal coordinate analysis (PCoA) plots in phyloseq. The effects of sex, treatment, and their interaction on the microbial community composition were assessed by two-way PERMANOVA using the adonis2 function in the vegan package (999 permutations) with the model Distance ~ Sex × Treatment. Analyses were conducted separately using Bray–Curtis, weighted UniFrac, and unweighted UniFrac distance matrices. To test the differences in microbial composition between groups, permutational multivariate analysis of variance (PERMANOVA) was performed using the adonis2 function in the vegan package [53]. Additionally, differential abundance analysis was performed using DESeq2 [54] within the phyloseq framework. Counts were modelled using a negative binomial generalised linear model, and statistical significance was assessed using the Wald test. Features were considered differentially abundant based on an FDR-adjusted *p* value < 0.05 and an absolute log2 fold change ≥ 2.

## 3. Results

### 3.1. α-Diversity Is Not Altered by Maternal Metformin Exposure

**Figure 1:**
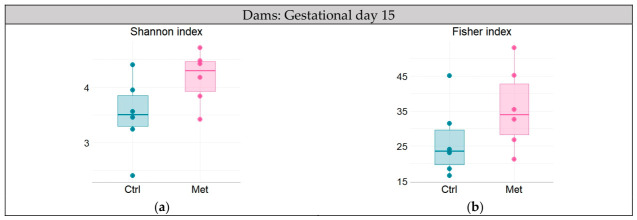
α-diversity of dams at GD15 measured by (**a**) Shannon and (**b**) Fisher metrics (Ctrl: control group, Met: metformin group). Each box indicates the median and interquartile range (IQR) from the 1st and 3rd quartiles. Whiskers extend to the minimum and maximum values within 1.5 times the IQR. Dots indicate individual values.

In dams at GD15, α-diversity was comparable between the control and metformin-treated groups, as assessed by both the Shannon index (*p* = 0.093) and Fisher’s alpha (*p* = 0.128) (Figure 1a,b). Consistently, other α-diversity metrics, including richness-based indices (ACE, Chao1, and Observed) and the evenness-based Simpson index, showed no significant differences between the two groups (Supplementary Table S1).

**Figure 2:**
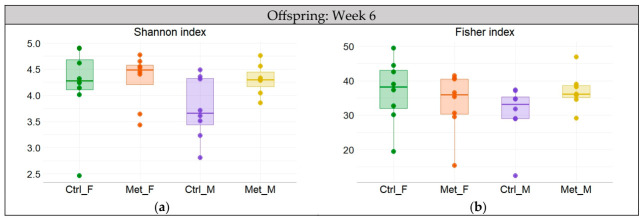
α-diversity of offspring at week 6 measured by (**a**) Shannon and (**b**) Fisher metrics (Ctrl: control group, Met: metformin group, F: female, M: male). Each box indicates the median and interquartile range (IQR) from the 1st and 3rd quartiles. Whiskers extend to the minimum and maximum values within 1.5 times the IQR. Dots indicate individual values.

Similarly, in offspring at 6 weeks of age, α-diversity did not differ significantly between maternal metformin exposure or sex. Shannon diversity and Fisher’s alpha showed no significant differences (Figure 2a,b), and this was consistent across additional diversity metrics (Supplementary Table S2).

### 3.2. Maternal Metformin Exposure Alters β-Diversity in Dams and Offspring

**Figure 3:**
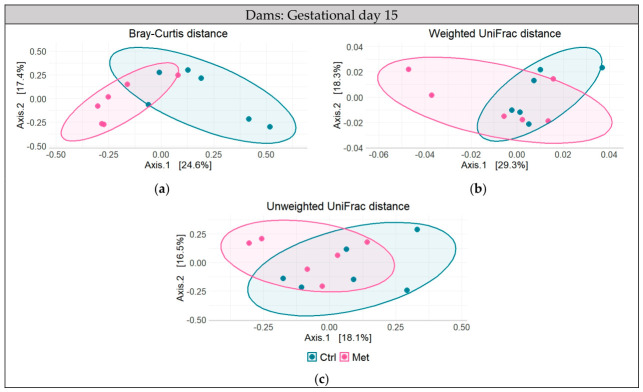
β-diversity ordination plot in dams at GD15 measured by (**a**) Bray–Curtis, (**b**) weighted, and (**c**) unweighted Unifrac PCoA (Ctrl: control group, blue; Met: metformin group, pink). Distribution of each group is outlined in blue and pink, respectively.

In dams, β-diversity differed significantly between the control and metformin-treated groups based on the Bray–Curtis dissimilarity (*p* = 0.0112), whereas no significant differences were observed using weighted (*p* = 0.085) or unweighted UniFrac metrics (*p* = 0.142) (Figure 3a–c).

**Figure 4:**
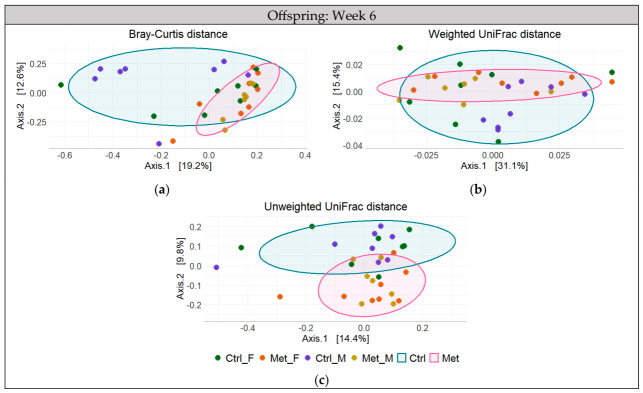
β-diversity ordination plot in offspring at week 6 measured by (**a**) Bray–Curtis, (**b**) weighted, and (**c**) unweighted UniFrac PCoA (Ctrl: control group, Met: metformin group, F: female, M: male). Individual points of Ctrl_F (green), Met_F (orange), Ctrl_M (purple), and Met_M (yellow) and main distributions of Ctrl (blue) or Met (pink) are outlined.

At 6 weeks of age, maternal metformin exposure significantly altered the β-diversity in male offspring, as assessed by the Bray–Curtis dissimilarity (*p* = 0.0060) and weighted UniFrac (*p* = 0.0036), but not in female offspring. The unweighted UniFrac also revealed significant differences between control and metformin-exposed offspring in both males (*p* = 0.0066) and females (*p* = 0.0146). No statistical significance between sexes in the same treatment was detected (Figure 4a–c). Additionally, the sex-by-treatment interaction was found to be significant in the weighted UniFrac analysis (R^2^ = 0.076, F = 2.445, *p* = 0.0240), whereas no significant interaction was observed using the Bray–Curtis or unweighted UniFrac distances (Supplementary Table S3).

### 3.3. Alterations in Microbial Composition Following Maternal Metformin Exposure

#### 3.3.1. Dams at Gestational Day 15

**Figure 5:**
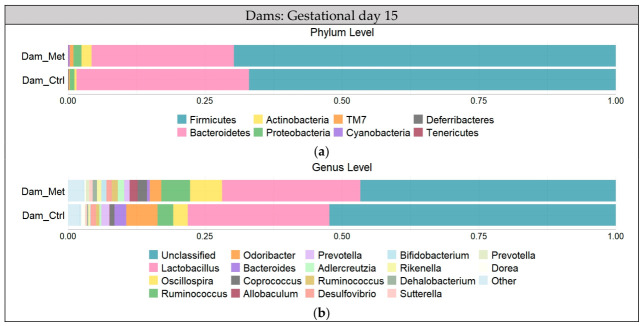
Relative abundance in dams on gestational day 15. Stacked bar charts show the relative abundances of amplicon sequence variants (ASVs) at the (**a**) phylum and (**b**) genus level (Ctrl: control group, Met: metformin group).

At the phylum level, metformin-treated dams exhibited increased relative abundances of Actinobacteria, Proteobacteria, and Cyanobacteria; reduced Bacteroidetes; and comparable levels of Firmicutes relative to controls (Actinobacteria: 1.84% vs. 0.37%; Proteobacteria: 1.48% vs. 0.87%; Cyanobacteria: 0.22% vs. 0.02%; Bacteroidetes: 25.97% vs. 31.48%; Firmicutes: 69.74% vs. 67.00%). Consequently, the Firmicutes-to-Bacteroidetes (F/B) ratio increased from 2.13 in controls to 2.69 following metformin treatment (Figure 5a; Supplementary Table S4).

At the genus level, metformin treatment was associated with an increased relative abundance of Actinobacteria-associated genera, including *Bifidobacterium*, *Adlercreutzia*, and *Olsenella*. Within Firmicutes, several SCFA-associated genera were enriched, including *Allobaculum*, *Roseburia*, *Coprococcus*, *Oscillospira*, *Ruminococcus*, and *Gemmiger*, whereas *Butyricicoccus* was reduced. In contrast, the opportunistic genus *Clostridium* and non-butyrate-producing genera (*Dorea* and *Candidatus* Arthromitus) showed decreased abundances, while *Anaerotruncus* and *Lactobacillus* remained stable. Within Proteobacteria, *Sutterella* was increased, *Desulfovibrio* was reduced, and *Bilophila* and *Acinetobacter* showed no clear differences between groups. Finally, several Bacteroidetes-associated genera, including *Bacteroides*, *Odoribacter*, and *Alistipes*, exhibited reduced relative abundances following metformin treatment (Figure 5b; Supplementary Table S5).

#### 3.3.2. Female Offspring at Week 6

**Figure 6:**
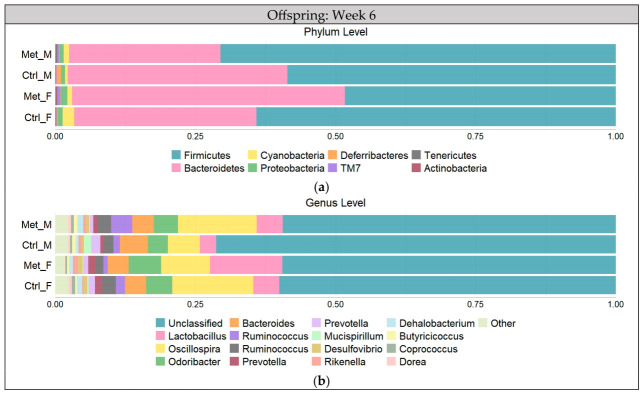
Relative abundance in offspring at week 6. Stacked bar charts show the relative abundances of amplicon sequence variants (ASVs) at the (**a**) phylum and (**b**) genus level. Group-level data by sex and control/metformin exposure (Ctrl: control group, Met: metformin group, F: female, M: male).

At the phylum level, metformin-exposed female offspring exhibited increased relative abundances of Actinobacteria, Proteobacteria, and Bacteroidetes compared with controls (0.27% vs. 0.11%, 1.19% vs. 0.92%, and 48.64% vs. 32.53%, respectively), while Firmicutes and Cyanobacteria were reduced (48.38% vs. 64.13% and 0.83% vs. 2.03%, respectively). Consequently, the F/B ratio decreased from 1.97 in controls to 0.99 following metformin exposure (Figure 6a; Supplementary Table S4).

At the genus level, metformin exposure was associated with an increased relative abundance of several Actinobacteria- and Proteobacteria-associated genera, including *Adlercreutzia*, *Olsenella*, *Desulfovibrio*, *Sutterella*, and *Acinetobacter*, while *Bilophila* and *Bifidobacterium* remained comparable between groups. Within Firmicutes, several butyrate-producing genera, including *Roseburia*, *Coprococcus*, *Oscillospira*, *Ruminococcus*, *Gemmiger*, and *Clostridium*, showed reduced abundances, whereas *Butyricicoccus* and *Allobaculum* did not differ between groups. Similar patterns were observed among minor or non-butyrate-producing genera, including *Dorea*, *Dehalobacterium*, and *Anaerotruncus*, while *Lactobacillus* was increased. Within Bacteroidetes, no consistent pattern was observed at the genus level, despite the overall increase at the phylum level (Figure 6b; Supplementary Table S5).

#### 3.3.3. Male Offspring at Week 6

At the phylum level, metformin-exposed offspring exhibited higher relative abundances of Firmicutes (70.54% vs. 58.59%) and Cyanobacteria (0.98% vs. 0.43%) compared with controls, while Bacteroidetes was reduced (26.98% vs. 39.22%). In contrast, Actinobacteria (0.08% vs. 0.14%) and Proteobacteria (0.77% vs. 0.77%) remained comparable between groups. Consequently, the F/B ratio increased from 1.49 in controls to 2.61 following metformin exposure (Figure 6a; Supplementary Table S4).

At the genus level, no consistent pattern was observed among taxa within Actinobacteria and Proteobacteria. Within Bacteroidetes, several commonly detected genera, including *Bacteroides*, *Prevotella*, and *Rikenella*, showed reduced abundances, whereas *Odoribacter* and *Alistipes* did not differ between groups. In contrast, most abundant genera within Firmicutes were increased in metformin-exposed offspring, including *Lactobacillus*, *Roseburia*, *Coprococcus*, *Oscillospira*, *Ruminococcus*, *Clostridium*, *Butyricicoccus*, *Allobaculum*, *Dorea*, and *Dehalobacterium* (Figure 6b; Supplementary Table S5).

### 3.4. Differential Abundance Following Maternal Metformin Exposure

#### 3.4.1. Dams at Gestational Day 15

**Figure 7:**
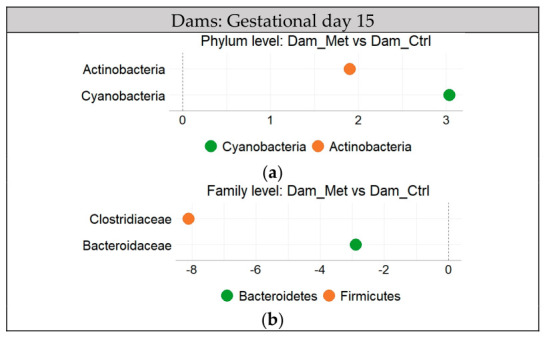
Key differentially abundant taxa in dams at gestational day 15 at the (**a**) phylum and (**b**) family level (Ctrl: control group, Met: metformin group). Only taxa that had a magnitude of change |log2FC| ≥ 2 with statistical significance (adjusted *p* value < 0.05) are shown in the plot, with the log2 fold change determined using DESeq2.

The differential abundance analysis using DESeq2 largely reflected the patterns observed in the relative abundance analyses. At the phylum level, Actinobacteria and Cyanobacteria were significantly enriched in metformin-treated dams, with log2 fold changes (log2FC) of 1.901 and 3.035, respectively (Figure 7a). At the family level, significant reductions were identified in Bacteroidaceae and Clostridiaceae following metformin exposure (log2FC = −2.894 and −8.102) (Figure 7b). No significant difference was found at the genus level.

#### 3.4.2. Offspring at Week 6

**Figure 8:**
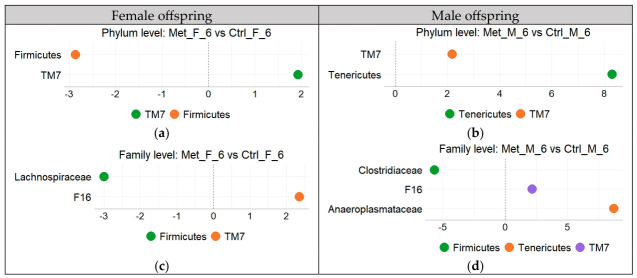
Key differentially abundant taxa in 6-week-old offspring at the (**a**,**b**) phylum and (**c**,**d**) family level (Ctrl: control group, Met: metformin group, F: female, M: male). Only taxa that had a magnitude of change |log2FC| ≥ 2 with statistical significance (adjusted *p* value < 0.05) are shown in the plot, with the log2 fold change determined using DESeq2.

At the phylum level, metformin exposure was associated with a significant reduction in Firmicutes and the enrichment of TM7 (Candidatus Saccharibacteria) in female offspring (log2FC = −2.86 and 1.93, respectively). In male offspring, significant increases were observed in the low-abundance phyla TM7 and Tenericutes (also referred to as Mycoplasmatota) (log2FC = 2.19 and 8.30, respectively) (Figure 8a,b).

At the family level, F16 was significantly enriched in both female and male offspring following metformin exposure (log2FC = 2.35 in females and 2.15 in males). In females, Lachnospiraceae was significantly reduced (log2FC = −3.00), whereas, in males, Anaeroplasmataceae was significantly increased (log2FC = 8.71) and Clostridiaceae was significantly decreased (log2FC = −5.69) (Figure 8c,d).

A sensitivity analysis excluding Cyanobacteria revealed minimal changes in the overall results regarding differential abundance in dams and offspring of both sexes (Supplementary Figure S2a–f).

## 4. Discussion

This study demonstrates that maternal metformin exposure during healthy murine pregnancy reshapes the maternal gut microbiota and induces distinct, sex-specific alterations in offspring microbial composition. In a non-dysbiotic setting, metformin did not broadly disrupt diversity but instead selectively remodelled the community structure, with divergent downstream effects on the early-life microbial composition. These findings highlight the context-dependent nature of metformin–microbiome interactions and underscore the sensitivity of the developing microbiome to maternal metabolic perturbation.

### 4.1. Metformin Selectively Remodels Dominant Microbial Taxa in Dams

In dams, metformin primarily influenced the relative abundance of dominant taxa rather than global community diversity, consistent with a targeted ecological effect. These changes largely aligned with established features of late pregnancy in human and mouse models, including the expansion of Actinobacteria [55,56,57,58] and an increased F/B ratio [59,60]. In particular, the enrichment of Actinobacteria—a characteristic of healthy pregnancy and associated with genera such as *Bifidobacterium*—supports the notion that metformin may reinforce, rather than disrupt, pregnancy-adapted microbial states [55,56,57,58].

Similarly, the elevated F/B ratio observed here, mainly driven by a reduction in Bacteroidetes, mirrors trends in murine pregnancy and likely reflects a physiological adaptation [59,60,61,62] rather than dysbiosis, despite its contrasting interpretation in non-pregnant metabolic disease contexts in human studies [63,64,65,66]. Within Firmicutes, the enrichment of butyrate-associated taxa further suggests a shift towards enhanced SCFA production, a hallmark of metabolic adaptation during pregnancy [67,68,69]. Despite the lack of DESeq2 significance at the genus level for beneficial Actinobacteria and butyrate producers after correction, their coordinated directionality suggests subtle yet biologically consistent restructuring. In contrast, the reduction in Clostridiaceae, including the opportunistic genus *Clostridium*, linked to adverse outcomes, may represent a beneficial effect of metformin on the maternal microbial environment [70,71].

### 4.2. Maternal Metformin Exposure Drives Broad Taxonomic Remodelling in Male Offspring

In male offspring, metformin exposure was associated with the broad restructuring of the gut microbiota, as evidenced by the consistent differences in β-diversity across multiple metrics despite preserved α-diversity. This pattern indicates community-wide reorganisation involving both dominant and low-abundance taxa, consistent with the heightened plasticity of the developing microbiome. Compared with the more targeted effects observed in dams, these findings suggest that early-life microbial assembly is particularly susceptible to systemic perturbations, with effects extending across multiple components of the microbial community.

A notable aspect of this response was the enrichment of low-abundance taxa, including members of Tenericutes and TM7, alongside related lineages such as Anaeroplasmataceae and F16. Although these taxa contribute minimally to overall biomass, they are increasingly recognised as potentially influential components of microbial networks. For example, *Anaeroplasma* has been considered as an indicator of ageing mice or as a potential anti-inflammatory probiotic [72,73], while its negative association with offspring body weight appears inconsistent across physiological contexts [74]. Similarly, TM7 (*Saccharibacteria*) has been reported in dysbiotic and inflammatory conditions including periodontal disease, mucosal infections, and gastrointestinal inflammation, where it may modulate host immune responses through direct attachment to and interaction with other bacterial cells [75,76,77]. These observations highlight the challenge of inferring function from taxonomic shifts alone and emphasise the context-dependent roles of rare taxa.

In parallel, the reduction in Clostridiaceae in both dams and male offspring suggests a conserved response to metformin that has been observed in other metabolic intervention studies [78,79,80,81]. However, the functional implications of this shift remain complex. While some *Clostridium* species are associated with dysbiosis and opportunistic infection, others contribute to early colonisation and host metabolic processes, including the utilisation of complex substrates such as human milk oligosaccharides and the modulation of inflammation [82,83]. This functional heterogeneity indicates that such changes are likely to reflect microbial restructuring rather than a uniform functional consequence.

At the level of dominant taxa, male offspring exhibited a shift toward Firmicutes expansion, accompanied by reduced Bacteroidetes and an elevated F/B ratio. This pattern contrasts with a study on non-pregnant humans with T2D, where metformin was associated with Bacteroidetes enrichment and reduced F/B ratios [84], highlighting the strong influence of the developmental and metabolic context on microbiome responses. The enrichment of Firmicutes-associated taxa implicated in SCFA production suggests a shift towards metabolic pathways relevant to immune maturation and host–microbe signalling during early life [85,86]. However, in the absence of direct functional measurements, it remains unclear whether these compositional shifts translate into altered metabolic output.

### 4.3. Metformin Induces Distinct Bacteroidetes-Dominant Shifts in Female Offspring

In contrast to males, female offspring exhibited a more selective microbial response characterised by constrained restructuring and a distinct compositional trajectory. Rather than broad microbial remodelling, metformin exposure appeared to preferentially affect low-abundance taxa, including the enrichment of TM7 and related lineages, suggesting the differential sensitivity of niche populations.

Despite this more limited restructuring, female offspring displayed a marked shift towards Bacteroidetes dominance, representing a divergence from both dams and male offspring. This pattern is consistent with early-life microbiota development in murine models, where the expansion of Bacteroidetes, often driven by S24–7 taxa, is associated with microbial maturation [87]. The reduction in Firmicutes-associated taxa, including Lachnospiraceae and related genera (*Roseburia*, *Coprococcus*, *Gemmiger*), further supports this shift. While these taxa are important early colonisers, their overrepresentation has also been linked to metabolic dysfunction in human and experimental models [88,89,90,91,92,93,94], indicating that their relative depletion may reflect the rebalancing of microbial functions rather than a detrimental effect.

In parallel, the enrichment of beneficial taxa such as *Lactobacillus* suggests a potential compensatory or protective response. Lactobacillus plays a well-established role in early-life microbial colonisation, supporting gut barrier integrity, immune regulation, and resistance to inflammatory conditions [95,96,97,98]. The coexistence of reduced Firmicutes diversity alongside the enrichment of specific beneficial taxa highlights the complexity of interpreting microbiome changes during early development.

Together, these findings suggest that maternal metformin exposure redirects microbiome development along sex-specific trajectories. While males exhibit widespread ecological restructuring, females show more targeted compositional shifts, underscoring the importance of the host biological context in shaping microbiome responses.

### 4.4. Host Metabolic and Developmental Context Shapes Microbiome Responses to Metformin

Metformin’s microbiome effects are likely mediated through its impact on host intestinal metabolism. By increasing intestinal glucose uptake and enhancing glycolytic activity, metformin alters luminal metabolite profiles, including lactate and pyruvate, which may favour specific microbial groups [99,100,101,102,103,104]. This altered environment may promote lactate-producing taxa and support cross-feeding interactions with SCFA-producing Firmicutes [105,106,107,108]. In contrast, changes in substrate availability may differentially affect Bacteroidetes, although responses appear context-dependent [109,110,111]. A cross-sectional study reported that metformin increased mucin-degrading *Akkermansia muciniphila* and several SCFA-producing bacteria in adults with T2D, suggesting its role in modulating mucin dynamics and intestinal niches favouring mucin degraders and SCFA producers [35,112]. Moreover, dams receiving metformin directly through drinking water displayed a marked increase in the F/B ratio and Actinobacteria, which was actively involved in mucin degradation [113,114,115,116]. Although metformin has been shown to decrease the elevated F/B ratio in obese and diabetic mouse models [117,118,119], our observation of an increased F/B ratio in healthy, chow-fed female mice indicates that metformin’s impact on microbial profiles is strongly context-dependent, varying with the host metabolic state and baseline microbial composition.

The effect of metformin on bile acid levels may also contribute to microbial shifts. In this study, the reduction in Bacteroidetes observed in metformin-treated dams is consistent with findings in individuals with newly diagnosed T2D, where metformin treatment reduced *Bacteroides fragilis* and was associated with a selective increase in the intestinal bile acid glycoursodeoxycholic acid [120]. Interactions between bile acids and the gut microbiota are bidirectional, and previous studies have reported associations between reduced secondary bile acids and lower relative abundances of taxa such as *Bifidobacterium* and *Clostridium* clusters [121]. Together, these findings suggest that the increased abundance of Actinobacteria and butyrate-producing *Clostridium* clusters observed in this study may be linked to alterations in bile acid metabolism, although the directionality of these relationships remains context-dependent. This interpretation is supported by evidence that metformin treatment can enrich bile acid- and SCFA-associated bacteria alongside increases in circulating secondary bile acids [122].

Importantly, maternal metformin treatment may result in the direct exposure of the offspring to metformin, as the drug is known to cross the placenta and is excreted into breast milk [123,124,125]. Additionally, mouse pups typically begin consuming water and solid food during the third postnatal week, with the transition from maternal milk to independent feeding occurring progressively before weaning [126]. However, in previous studies, infant exposure remained minimal (approximately 0.13–1.28 mg/day), with serum metformin concentrations undetectable in nursing infants and no reported effects on infant blood glucose levels [125,127,128]. In our study, offspring faecal samples for microbiome analysis were collected at six weeks of age, following a three-week period after the cessation of metformin exposure. Therefore, the offspring gut microbiota may not be expected to exhibit shifts comparable to those observed in dams, given the indirect exposure during lactation and the washout period prior to sample collection. Notably, the microbial responses to maternal metformin exposure in offspring did not only differ from those observed in dams but also showed sex-specific patterns. Consistent with this, studies in high-fat-diet-induced type 2 diabetes mouse models have shown that metformin exerts more pronounced effects on metabolic markers in males, whereas the microbial community composition is more markedly altered in females, highlighting sex-dependent responses in microbiome remodelling [129,130,131]. Nevertheless, within the same maternal treatment group in our study, sex-associated differences were modest, with no significant difference detected across taxonomic levels.

## 5. Limitations

Several limitations of this study should be acknowledged. First, maternal metformin treatment during healthy pregnancy is not widely representative of current clinical practice, and treatment for conditions such as GDM or PMOS is typically discontinued at delivery. In this study, metformin administration across both pregnancy and lactation may have complicated the interpretation of offspring microbiota outcomes, as offspring were exposed to metformin through distinct routes—directly during gestation and indirectly during lactation. Second, individual water consumption was not monitored, precluding confirmation of the actual daily metformin intake by each dam. Based on published estimates of water consumption in C57BL/6J mice, metformin-treated dams were expected to receive approximately 1100 mg/kg/day [132]; however, variations in water intake may have resulted in differences in actual drug exposure. Moreover, serum metformin concentrations were not measured, preventing confirmation of systemic drug exposure and assessments of inter-animal variability. Consequently, any association between metformin administration, circulating drug levels, and microbiota outcomes should be interpreted cautiously. In addition, although litter sizes were standardised to six pups to reduce variability in maternal resource allocation and early-life nutritional exposure, the potential influence of litter-specific factors cannot be entirely ruled out. Pups within the same litter shared a common maternal environment and were exposed to similar early microbial communities, and these shared factors may have contributed, at least in part, to the observed variations in offspring gut microbiota composition.

Furthermore, a relatively large proportion of reads remained unclassified at the genus level. This likely reflects the limited taxonomic resolution of V3–V4 16S rRNA gene sequencing and the incompleteness of current reference databases. As a result, some taxa could not be confidently assigned to the genus level despite successful classification at higher taxonomic ranks, and the genus-level findings should therefore be interpreted with appropriate caution. Moreover, Cyanobacteria detected in murine faecal 16S rRNA sequencing datasets may represent chloroplast-derived or environmental contaminants rather than bona fide gut bacteria. Excluding Cyanobacteria from the analysis resulted in minimal changes to the differential abundance results, suggesting that their presence did not substantially affect the study conclusions. However, taxonomic assignments to this phylum should be interpreted cautiously. In addition, although 16S rRNA sequencing of faecal DNA does not fully capture microbial communities across all regions of the gastrointestinal tract, faecal sampling from spontaneous defecation represents a non-invasive and widely accepted approach. The microbial density in the colon is the most abundant, being more than one million times higher than the density in other regions [133]. Furthermore, in a healthy, non-dysbiotic system, metformin-induced microbiome modulation is expected to be subtle and broadly distributed across multiple taxa, rather than driven by large shifts in individual genera. Such diffuse changes may be difficult to detect following statistical testing and correction, potentially masking biologically relevant effects. Finally, although metformin treatment was associated with changes in the relative abundance of taxa reported to produce short-chain fatty acids (SCFAs), faecal SCFA concentrations were not measured. Therefore, it was not possible to determine whether these compositional changes translated into altered SCFA production or availability. Future studies incorporating direct metabolomic analyses, such as gas chromatography–mass spectrometry-based SCFA quantification, are needed to confirm the functional significance of the observed microbiota alterations.

## 6. Conclusions

This study is among the first to highlight metformin’s impact on gut microbial remodelling during healthy pregnancy and demonstrate a sex-specific interaction between maternal metformin exposure and early-life microbial profiles in offspring at 6 weeks of age. Metformin is likely to promote beneficial changes associated with healthy pregnancy in the maternal microbial composition, despite the presence of some potentially unfavourable effects. Notably, metformin exposure also induced divergent microbiota profiles in offspring between sexes, with a greater number of beneficial microbial shifts observed. Given the modifiable and accessible nature of the gut microbiome, further investigation—particularly in human cohorts and using higher-resolution approaches—is required to elucidate the underlying mechanisms and functional roles of relevant microbial alterations. Such insights may ultimately inform maternal and postnatal care by improving pregnancy outcomes and guiding the development of preventive interventions.

## Data Availability

The original contributions presented in this study are included in the article/Supplementary Materials. Further inquiries can be directed to the corresponding author.

## Supplementary Materials

The following supporting information can be downloaded at https://www.mdpi.com/article/10.3390/biom16071017/s1, Supplementary Figure S1: Data distribution; Supplementary Figure S2: Sensitivity analysis excluding Cyanobacteria; Supplementary Table S1: α-diversity in dams at GD15; Supplementary Table S2: α-diversity in 6-week-old offspring; Supplementary Table S3: Sex–treatment interaction in 6-week-old offspring; Supplementary Table S4: Relative abundance (%) at phylum level and F/B ratio; Supplementary Table S5: Relative abundance (%) of top 30 most abundant genera.

## Abbreviations

The following abbreviations are used in this manuscript:

GDM	Gestational diabetes mellitus
T2D	Type 2 diabetes
PCOS	Polycystic ovary syndrome
PMOS	Polyendocrine metabolic–ovarian syndrome
SCFA	Short-chain fatty acid
PCR	Polymerase chain reaction
PERMANOVA	Permutational multivariate analysis of variance
FDR	False discovery rate
p_adj_	Adjusted *p* value
PCoA	Principal coordinate analysis
DESeq2	Differential Expression Analysis for Sequencing Data (version 2)
log2FC	Log base 2 fold change
a diversity	Alpha diversity
b diversity	Beta diversity
F/B	Firmicutes-to-Bacteroidetes
AGRF	Australian Genome Research Facility
GD	Gestational day (GD0.5, GD15)
F	Female
M	Male
Ctrl	Control treatment
Met	Metformin treatment
