# Supplementary material for: Maternal Metformin Exposure Induces Gut Microbial Shifts in Non-Diabetic Dams and Sex-Stratified Changes in Mouse Offspring
Source: Biomolecules. 2026 Jul 12;16(7):1017. doi: 10.3390/biom16071017 (PMC13406231; doi:10.3390/biom16071017)
Supplement: Supplementary file 1 [file biomolecules-16-01017-s001.zip › biomolecules-4409522-supplementary.pdf]

biomolecules
MDPI

Supplementary Materials

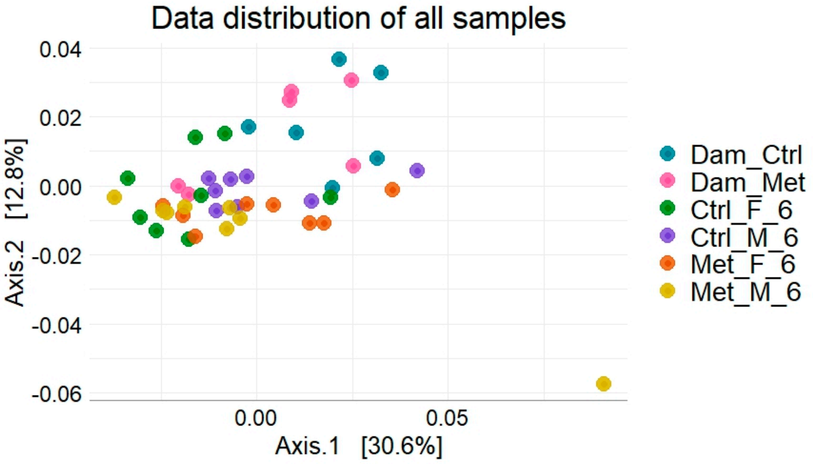

Mouse ID	*Group*	*Axis 1*	*Axis 2*	*MD2*	*p value*	*Outlier*
49	Met_M_6	0.091	-0.058	26.867	1.47E-06	True

Supplementary Figure S1. Data distribution. Outliers were identified using the Mahalanobis distance squared (MD^2) and a chi-square threshold ($p < 0.05$).

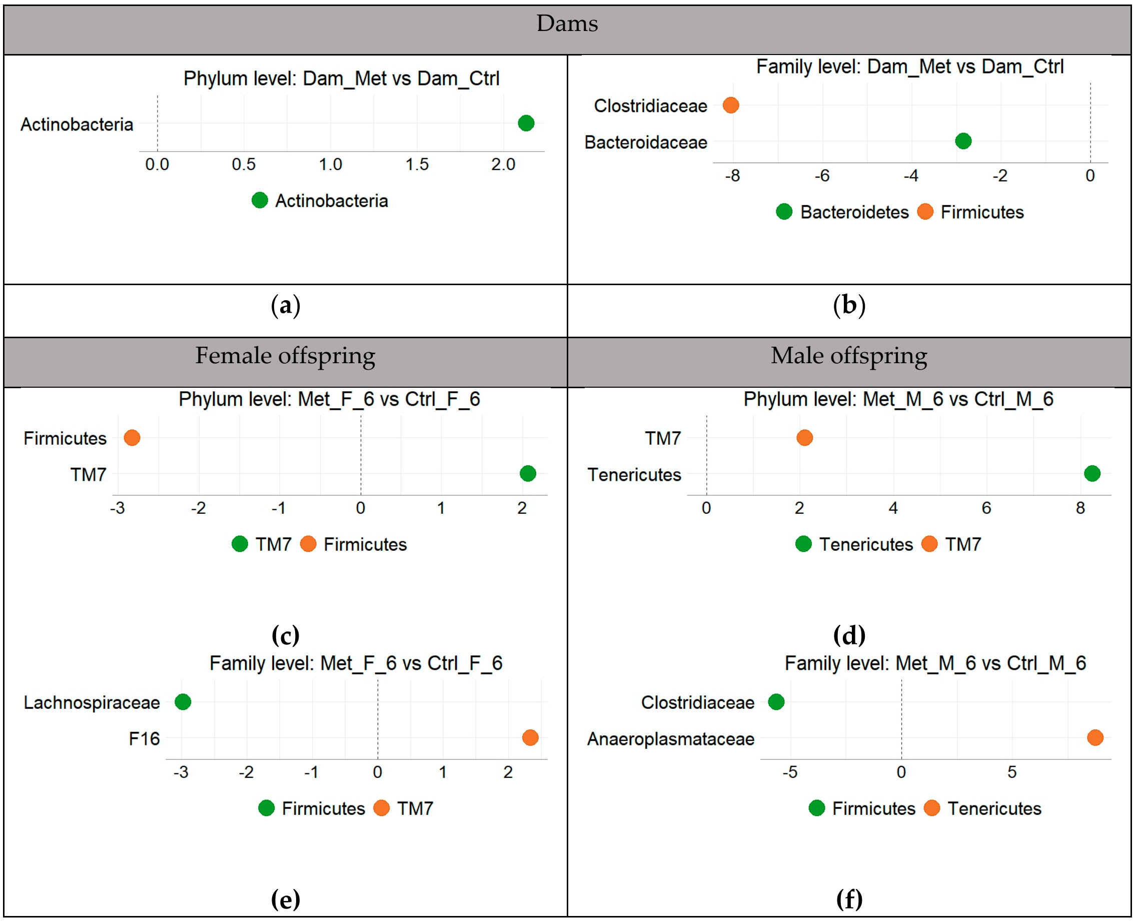

Supplementary Figure S2. Sensitivity analysis excluding Cyanobacteria. Key differentially abundant taxa in dams (a, b) and 6-week-old offspring (c-f) at phylum and family level (Ctrl: control group, Met: metformin group, F: female, M: male). Only taxa, which had the magnitude of change |log2FC| >= 2 with statistical significance (adjusted p value < 0.05), are shown in the plot, with log2 fold change determined using DESeq2.

Supplementary Table S1. α diversity in dams at GD15.

Measure	Dam_Ctrl	Dam_Met	p†
Shannon	3.50 ± 0.68	4.18 ± 0.48	0.093
Fisher	26.47 ± 10.52	35.75 ± 11.74	0.128
ACE	185.33 ± 70.70	247.83 ± 72.02	0.128
Chao1	185.33 ± 70.70	247.83 ± 72.02	0.128
Observed	185.33 ± 70.70	247.83 ± 72.02	0.128
Simpson	0.91 ± 0.05	0.95 ± 0.04	0.093

† Wilcoxon test

Supplementary Table S2. α diversity in 6-week-old offspring.

Measure	Ctrl_F	Met_F	p†	Ctrl_M	Met_M	p†
Shannon	4.20 ± 0.78	4.30 ± 0.49	0.875	3.75 ± 0.59	4.30 ± 0.30	0.118
Fisher	36.86 ± 9.41	33.75 ± 8.67	0.431	30.73 ± 8.09	37.08 ± 5.39	0.093
ACE	253.75 ± 65.04	234.62 ± 73.56	0.599	223.88 ± 70.15	265.71 ± 41.54	0.325
Chao1	253.75 ± 65.04	234.62 ± 73.56	0.599	223.88 ± 70.15	265.71 ± 41.54	0.325
Observed	253.75 ± 65.04	234.62 ± 73.56	0.599	223.88 ± 70.15	265.71 ± 41.54	0.325
Simpson	0.95 ± 0.05	0.97 ± 0.02	0.793	0.93 ± 0.05	0.97 ± 0.01	0.118

† Wilcoxon test

Supplementary Table S3. Sex-treatment interaction in 6-week-old offspring.

	Bray-Curtis			Weighted UniFrac			Unweighted UniFrac		
	R^2	F	p†	R^2	F	p†	R^2	F	p†
Sex	0.031	1.010	0.452	0.028	0.893	0.483	0.027	0.865	0.688
Treatment	0.084	2.685	0.001	0.059	1.903	0.051	0.080	2.538	0.001
Sex-Treatment	0.044	1.407	0.099	0.076	2.445	0.024	0.041	1.288	0.092

† Two-way Permanova

Supplementary Table S4. Relative abundance (%) at phylum level and F/B ratio.

Phylum	Dams		Offspring			
	Dam_Ctrl	Dam_Met	Ctrl_F	Met_F	Ctrl_M	Met_M
Firmicutes	67.00	69.74	64.13	48.38	58.59	70.54
Bacteroidetes	31.48	25.97	32.53	48.64	39.22	26.98
Cyanobacteria	0.02	0.22	2.03	0.83	0.43	0.98
Actinobacteria	0.37	1.84	0.11	0.27	0.14	0.08
Proteobacteria	0.87	1.48	0.92	1.19	0.77	0.77
Deferribacteres	0.10	0.01	0.16	0.10	0.75	0.05
TM7	0.16	0.69	0.07	0.55	0.10	0.34
Tenericutes	0.01	0.06	0.04	0.06	0.001	0.27
**F/B ratio**	**2.13**	**2.69**	**1.97**	**0.99**	**1.49**	**2.61**

Supplementary Table S5. Relative abundance (%) of top 30 abundant genera.

Genus	Dams		Offspring			
	Dam_Ctrl	Dam_Met	Ctrl_F	Met_F	Ctrl_M	Met_M
Unclassified	52.74	47.87	60.83	59.88	72.17	59.89
Lactobacillus	25.85	25.26	4.65	12.92	2.84	4.60
Oscillospira	2.61	5.86	14.43	8.66	5.71	14.07
Odoribacter	5.68	2.09	4.68	5.83	3.62	4.30

Ruminococcus	3.49	6.39	4.06	2.19	2.80	6.14
Bacteroides	2.13	0.45	3.79	3.75	4.99	3.82
Coprococcus	0.87	1.72	0.58	0.33	0.38	0.50
Allobaculum	0.07	1.49	0.04	0.06	0.01	0.02
Prevotella	1.60	1.60	2.40	2.18	2.25	1.46
Adlercreutzia	0.38	1.17	0.12	0.20	0.20	0.09
Mucispirillum	0.16	0.01	0.27	0.12	1.33	0.09
Desulfovibrio	1.03	0.95	0.70	0.86	0.44	0.64
Bifidobacterium	0.11	0.94	0.03	0.01	0.002	0.02
Rikenella	0.28	0.81	0.17	0.85	0.52	0.46
Dehalobacterium	0.25	0.77	1.00	0.67	0.51	0.99
Sutterella	0.26	0.61	0.28	0.42	0.33	0.17
Dorea	0.71	0.29	0.56	0.13	0.34	0.48
Roseburia	0.04	0.59	0.01	0.001	0.004	0.02
Butyricicoccus	0.67	0.14	0.27	0.36	0.64	0.69
Clostridium	0.58	0.29	0.33	0.10	0.34	0.44
Anaeroplasma	0.01	0.02	0.07	0.08	<0.001	0.45
Olsenella	0.08	0.22	0.04	0.11	0.04	0.01
Parabacteroides	0.02	0.04	0.24	0.11	0.23	0.27
Anaerotruncus	0.17	0.14	0.26	0.11	0.21	0.20
Alistipes	0.01	<0.001	0.10	<0.001	0.001	0.08
Bilophila	0.01	0.01	0.03	0.01	0.01	0.09
C. Arthromitus	0.10	<0.001	0.01	0.01	0.02	<0.001
Gemmiger	<0.001	0.03	0.01	<0.001	0.01	<0.001
Acinetobacter	0.04	0.03	<0.001	0.01	0.003	<0.001
Other	0.20	0.27	0.06	0.05	0.10	0.01

https://doi.org/10.3390/xxxxx
